# Dimeric and polymeric IgA, but not monomeric IgA, enhance the production of IL-6 by human renal mesangial cells

**DOI:** 10.1155/S0962935196000269

**Published:** 1996-06

**Authors:** T. J. F. Reterink, W. E. M. Schroeijers, L. A. van Es, M. R. Daha

**Affiliations:** 1 Department of Nephrology Leiden University Hospital C3P P.O. Box 9600 Leiden 2300 RC The Netherlands

## Abstract

Depositions of IgA in the renal glomerular mesangial area are a hallmark of IgA nephropathy, and are thought to be crucial for the onset of inflammation processes in IgA nephropathy. In this report we show that human mesangial cells (MC) *in vitro* bind IgA and that binding of IgA enhances the production of IL-6 by MC. Furthermore we show that the size of IgA is crucial in its capability to enhance IL-6 production. Monomeric IgA does not affect basic IL-6 production, whereas dimeric and polymeric IgA enhance IL-6 production up to 3- to 9-fold respectively. Additional studies demonstrate that enhanced IL-6 production by MC is not accompanied by increased proliferation of human mesangial cells, a finding which is distinct from that found with rat mesangial cells. Taken together, these fmdings suggest that deposition of dimeric and polymeric IgA in the mesangial area of human kidneys in IgA nephropathy may amplify local inflammation.


Mediators of Inflammation, 5, 191–195 (1996)

### Dimeric and polymeric IgA, but not monomeric IgA, enhance the production of IL-6 by human renal mesangial cells

T. J. F. Reterink,[CA] W. E. M. Schroeijers, L. A. van Es and M. R. Daha

Department of Nephrology, Leiden University Hospital, C3P, P.O. Box 9600, 2300 RC Leiden, the Netherlands

[CA]Corresponding Author

**Depositions of IgA in the renal glomerular mesangial area are a hallmark of IgA nephropathy, and are thought to be crucial for the onset of inflammation processes in IgA nephropathy. In this report we show that human mesangial cells (MC) *in vitro* bind IgA and that binding of IgA enhances the production of IL-6 by MC. Furthermore we show that the size of IgA is crucial in its capability to enhance IL-6 production. Monomeric IgA does not affect basic IL-6 production, whereas dimeric and polymeric IgA enhance IL-6 production up to 3- to 9-fold respectively. Additional studies demonstrate that enhanced IL-6 production by MC is not accompanied by increased proliferation of human mesangial cells, a finding which is distinct from that found with rat mesangial cells. Taken together, these findings suggest that deposition of dimeric and polymeric IgA in the mesangial area of human kidneys in IgA nephropathy may amplify local inflammation.**

**Key words**: IgA, Interleukin 6, Renal mesangial cells

### Introduction

Immunoglobulin A (IgA) is the predominant immunoglobulin in human secretions and the second most important immunoglobulin in the circulation on a quantitative basis.[1,2] Depositions of IgA in the glomerular mesangial area of the kidney, as found in IgA nephropathy, are thought to play a crucial role in the inflammatory processes in this disease. The deposited IgA is mainly of the IgA1 subclass[3] and is thought to be derived from the circulation.[4] Also co-depositions of IgG and complement factors, such as complement component 3 (C3) are routinely seen in renal biopsies of patients with IgA nephropathy.[5] Elevated serum levels of IgA,[6] increased production of IgA1,[7] and a hyperresponse for IgA1 after vaccination[7] is found in patients with IgA nephropathy. The mechanisms of IgA deposition in the kidney are still unclear. However, a delayed clearance of IgA has been suggested.[8] Recently, a specific receptor for IgA was described in rat and human renal glomerular mesangial cells (CD89).[9] It is not known whether CD89 plays a role in IgA deposition in the kidney during IgA nephropathy. It is interesting that phagocytic blood cells of patients with IgA nephropathy seem to be hampered in CD89-mediated clearance of IgA.[10]

Mesangial depositions of IgA have been associated with increased levels of IL-6 in the urine of patients with IgA nephropathy.[6] IL-6 is a potent, multifunctional cytokine, which has multiple biological function activities on a wide variety of tissues and cells, such as B- and T-lymphocytes, myeloma cells, haematopoietic stem cells, hepatocytes, fibroblasts[11] and rat glomerular mesangial cells.[12] It was shown that IL-6 is produced by a variety of cells, such as macrophages, lymphocytes, fibroblasts and endothelial cells.[13–17] Also, it has been shown that MC are able to produce IL-6,[18–20] which acts as an autocrine growth factor for rat mesangial cells *in vitro*.[12,21]

In this study we demonstrate that human MC bind IgA and produce IL-6 upon stimulation with IgA *in vitro*. The degree of production of IL-6 is dependent on the size of the IgA. Only dimeric and polymeric IgA enhance IL-6 production by MC. The IgA-induced production of IL-6 is inhibitable by cycloheximide, which indicates *de novo* synthesis. Furthermore we show that human MC do not proliferate upon stimulation with IgA.

### Materials and Methods

*Cell culture:* Glomerular mesengial cells (MC) were cultured using glomeruli obtained from human foetal kidneys of 12–19 weeks of gestation, by mechanical dissociation and sequential sieving as described.[22,23] Approval for the use of foetal kidneys was obtained by informed consent and from the medical ethics committee of the hospital. After sieving, glomerular epithelial cells

© 1996 Rapid Science Publishers were removed by digestion with type 1A collagenase (Sigma, St Louis, MO, USA) for 20 min at 37°C. After washing, the resulting glomerular suspensions were resuspended in DMEM (Seromed, Biochrom, Berlin, Germany) supplemented with 20% heat-inactivated foetal calf serum (FCS) (Gibco, Breda, The Netherlands), plated onto charged plastic culture Primaria flasks (Falcon, Becton Dickinson, San Jose, CA) and cultured at 37°C in 5% $CO_2$. After outgrowth of the MC, the hillocks formed were lifted off the culture flasks and explanted into 24-well culture plates (Greiner, Alphen aan de Rijn, The Netherlands). MC growing out of the hillocks were subcultured in T25 or T75 culture flasks (Greiner). For the experiments MC were used between subculture 2 and 8.

*Isolation of IgA:* Human IgA was isolated by chromatography using DEAE Sephadex (Pharmacia, Uppsala, Sweden) anion exchange chromatography, and Sephacryl S-300 (Pharmacia) gel filtration as described.[24] Purity of the final IgA preparations was checked by SDS–polyacrylamide gel and ELISA for residual IgG and IgM. The IgA preparations were shown to be free of detectable IgG and IgM by ELISA.

Rat IgA was isolated as described,[25] briefly, IgA containing ascites from Lewis rats, inoculated intraperitoneally with $10^6$ viable LO-DNP-45 hybridoma cells, was collected and precipitated with a final concentration of 50% $(NH_4)_2SO_4$. The pellet was resuspended and dialysed against phosphate-buffered saline (PBS)-2 mM EDTA and IgA-anti-dinitrophenol (DNP) was affinity purified using a DNP-lysine-coupled Sepharose affinity column. After washing, anti-DNP-specific IgA was eluted from the column with 0.1 M DNP. After removal of free DNP by chromatography on Dowex 1 (1 × 2 – 400), the IgA containing fractions were pooled, concentrated and subjected to gel filtration chromatography on a Sephacryl S-300 column to yield monomeric, dimeric- and polymeric IgA. The purified IgA preparations were dialysed against PBS and were shown to be devoid of detectable amounts of IgA and IgM, as detected by ELISA.

*Radiolabelling of IgA:* Human dimeric IgA was radiolabelled with $^{125}I$ using Iodo-Beads (Pierce, Rockford, IL) according to the manufacturer's instructions. Non-incorporated iodine was separated from protein by gel filtration using Sephadex G25 (Pharmacia). The specific activity was 3 μCi/μg protein.

*Binding of IgA to MC:* After growing to subconfluency in 48-well plates, MC were washed three times with PBS/0.5% bovine serum albumin (BSA), and three wells were trypsinized to count the number of cells present at the beginning of the assay, using a Coulter Counter (Coulter Electronics, Mijdrecht, The Netherlands). Subsequently, MC were incubated for 16 h at 4°C with a dose response of iodinated human IgA in 300 μl DMEM/0.5%BSA in triplicate. After incubation, the wells were washed three times with cold PBS/0.5%BSA to remove non-bound radioactivity. After washing, 300 μl 1 M NaOH was added per well, to detach and solubilize the cells and subsequently, the amount of radioactivity was measured and calculated per μg/$10^5$ cells. All data were corrected for nonspecific binding.

*IL-6 production:* Subconfluent 48-well plates with MC were washed three times with phosphate buffered saline (PBS) and cultured for an additional 48 h in DMEM/0.5%FCS to induce a quiescent state. After washing with DMEM/0.5%FCS, fixed concentrations of IgA were added to the cells in DMEM/0.5%FCS in triplicate. As a positive control, MC were stimulated with 100 ng/ml LPS. After 72 h of stimulation, supernatants were harvested and assessed for IL-6 production in the B9-bioassay, using the Il-6-dependent murine hybridoma cell line B9,[20,26] kindly provided by Dr L. A. Aarden (CLB, Amsterdam, The Netherlands) in combination with the Cell Titer 96 assay (Promega, Leiden, The Netherlands) to measure proliferation. Serial dilutions of human recombinant IL-6 were used as a standard.

*Effect of cycloheximide on the production of IL-6 by MC:* After growing MC to subconfluency in 48-well plates, the cells were washed and incubated further, either in medium alone, or in medium containing 10 μg/ml human dimeric IgA, with or without 1 μg/ml cyclohexamide in triplicate. After 72 h, the supernatants were harvested and used in the B9 assay in serial dilutions of 1/20–1/640. Cycloheximide at concentrations of less than 5 ng/ml does not interfere with the B9 assay. To determine whether the cells remained viable during incubation with cycloheximide, the cells were washed with medium and incubated in medium alone for another 72 h. After this second incubation, the supernatants were harvested again, and assessed for IL-6 using the B9 assay.

*MC proliferation assay:* MC, subconfluently plated in 96- or 48-well plates, were washed three times with phosphate buffered saline (PBS) and cultured for an additional 48 h in DMEM/0.5% to induce a quiescent state. After washing with DMEM/0.5%FCS, fixed amounts of the various IgA preparations in DMEM/0.5%FCS were added in triplicate. As a positive control, 100 ng/ml LPS was used. After 7 days of stimulation, the cells were washed, trypsinized and counted by using a Coulter Counter.

### Results

To analyse the binding of IgA to MC, the MC were incubated at 4°C with a dose response of $^{125}$I-labelled human dimeric IgA for 16 h. Human dimeric IgA was able to bind to MC in a dose-dependent manner. Saturation was reached at approximately 10 µg/ml (Fig. 1).

To investigate whether MC are activated upon binding of IgA, MC were incubated at 37°C in triplicate wells for 72 h in medium (DMEM/10%FCS) alone, in medium supplemented with increasing concentrations of human dimeric IgA, or in medium containing 100 ng/ml LPS, as a positive control. After stimulation, the amount of IL-6 produced by the MC was measured in the B9 assay. Basal production of IL-6 by MC was 1286 ± 414 units IL-6 per $10^5$ cells. Culture of MC with human dimeric IgA resulted in up-regulation of IL-6 production in a dose-dependent fashion. At the highest concentration of 100 µg/ml, human dimeric IgA was able to induce a 2.5-fold up-regulation of 3171 ± 1400 units IL-6 per $10^5$ cells ($p <$ 0.05). LPS, as a positive control, induced a production of 6043 ± 2386 units IL-6 per $10^5$ cells (Fig. 2).

To investigate whether the production of IL-6 is dependent on the size of IgA, MC were stimulated in triplicate wells for 72 h with medium alone, 0.1, 10 and 100 µg/ml rat monomeric, dimeric, and polymeric IgA, or 100 ng/ml LPS. Polymeric IgA was able to induce a three-fold increase to 4086 ± 1443 units IL-6 per $10^5$ cells at a concentration of 10 µg/ml and a nine-fold increase to 11800 ± 300 units IL-6 per $10^5$ cells at a concentration of 100 µg/ml. Dimeric IgA was much less potent in inducing an increase of IL-6 production. At a concentration of 100 µg/ml, dimeric IgA induced a production of 3586 ± 1471 units IL-6 per $10^5$ cells, whereas monomeric IgA did not induce a significant increase in IL-6 production (Fig. 3).

To investigate whether the IL-6 produced by the MC upon IgA stimulation is due to *de novo* synthesis, MC were incubated in triplicate for 72 h in medium, or in medium supplemented with 10 µg/ml human dimeric IgA, both with or without cycloheximide (1 µg/ml), and assessed for IL-6. Cycloheximide was able to cause 97.3% inhibition of IgA-induced IL-6 release ($p <$ 0.002) (Fig. 4).

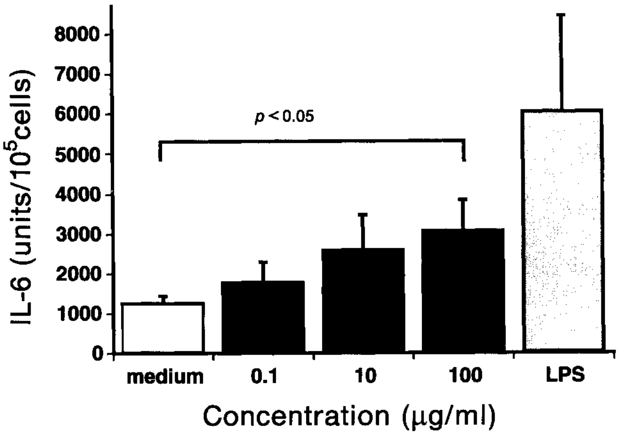

FIG. 2. Effect of increasing concentrations of human dimeric IgA on the production of IL-6 by MC *in vitro*. MC were incubated in triplicate with IgA for 72 h. After incubation the supernatants were assessed for IL-6. The effect of LPS, as a positive control, is also shown on the right.

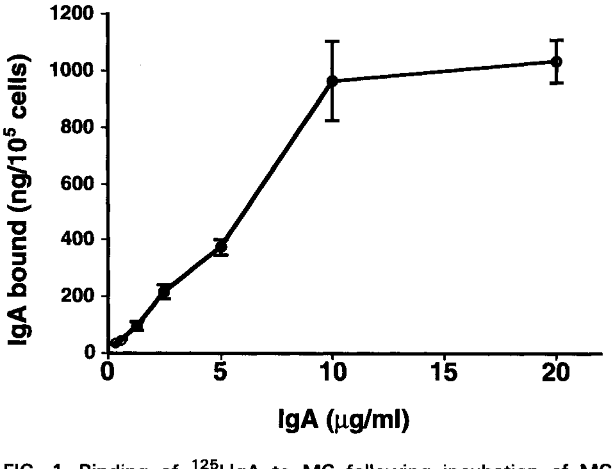

FIG. 1. Binding of $^{125}$I-IgA to MC following incubation of MC with increasing concentrations of $^{125}$I-labelled human dimeric IgA for 16 h at 4°C. One of four representative experiments is shown. Results are expressed as the mean ± S.E.M. of triplicate wells.

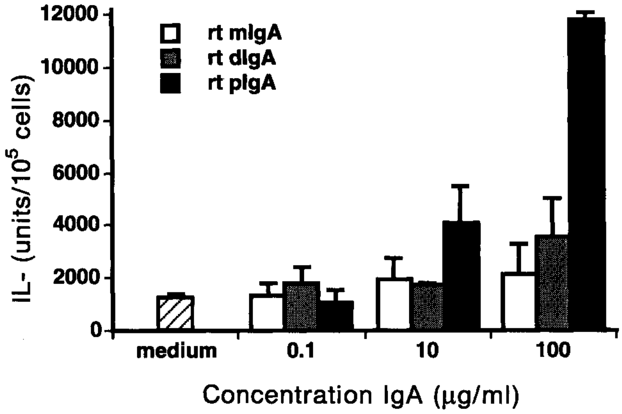

FIG. 3. Effect of increasing concentrations of rat monomeric, dimeric or polymeric IgA on the production of IL-6 by MC *in vitro*. MC were incubated with various IgA preparations in triplicate for 72 h. After incubation, the supernatants were harvested and assessed for IL-6. One of three representative experiments is shown. Results are expressed as the mean ± S.E.M.

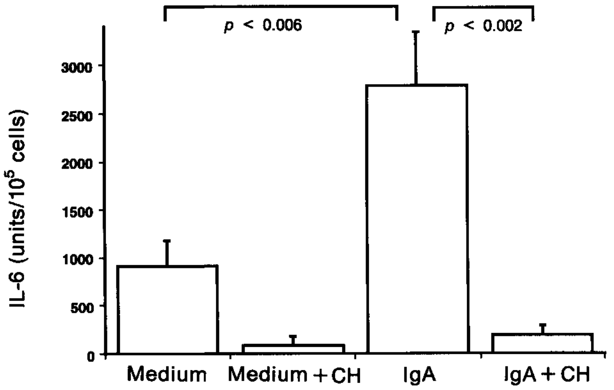

FIG. 4. Effect of cycloheximide on the production of IL-6 by MC *in vitro*. MC were incubated in triplicate with medium alone or medium supplemented with human dimeric IgA (10 μg/ml), with or without cycloheximide (1 μg/ml). After 72 h, the supernatants were harvested and assessed for IL-6. Results are expressed as the mean ± S.E.M.

It has been suggested that IgA is able to induce proliferation of rat MC *in vitro*. To investigate whether human MC also proliferate upon stimulation with IgA, MC were incubated in medium alone (DMEM/0.5%FCS) or with various preparations of IgA at a concentration of 10 μg/ml. As a positive control 100 ng/ml LPS was used. After 7 days of culture, the triplicate wells were trypsinized and the total cell number per well was determined by using the Coulter Counter. None of the IgA preparations was able to induce MC proliferation. In contrast, 100 ng/ml LPS was able to induce a relative proliferation of 2.24 ± 0.41 ($p < 0.05$) (Fig. 5).

### Discussion

Glomerular mesangial depositions of IgA in the kidney, together with IgG and complement components such as C3, are thought to be crucial for the onset of the inflammation process in IgA nephropathy. It has been shown that IgG binds to MC[27,28] and stimulates IL-6 production.[18,20] In this study, we show that human MC are capable of secreting *de novo* synthesized IL-6 after stimulation with IgA. The IL-6 production of the MC is strongly dependent on the size of the IgA. Polymeric rat IgA is able to increase the IL-6 production by MC up to nine-fold. Dimeric IgA is less potent in increasing IL-6 production by MC and only increased the basal production three-fold. In contrast with polymeric and dimeric IgA, monomeric IgA is not able to increase the IL-6 production.

It has been shown that IL-6 is an autocrine growth factor for rat MC.[12,21] Even though dimeric and polymeric IgA enhance the production of IL-6 by human MC, we did not observe proliferation of the cells. These findings are in agreement with the observation that human MC do not proliferate upon stimulation with IL-6[29,30] *in vitro*. In IL-6 transgenic mice, it was shown that high plasma concentrations of IL-6 are associated with mesangial proliferation.[31] In humans, however, it was found that urinary IL-6 levels do not correlate with mesangial proliferative glomerulonephritis.[32] Our finding, that MC fail to proliferate upon stimulation with IgA, is also in accord with these findings. Therefore, our *in vitro* data suggest that mesangial cell proliferation in humans, as found in IgA nephropathy, is presumably not due to the autocrine effect of IL-6 produced by MC upon stimulation with IgA. The precise mechanisms by which mesangial proliferation is induced remain unclear, suggesting that other mitogens, or combinations of different mitogens acting synergistically, induce the mesangial proliferation, as seen in IgA nephropathy.

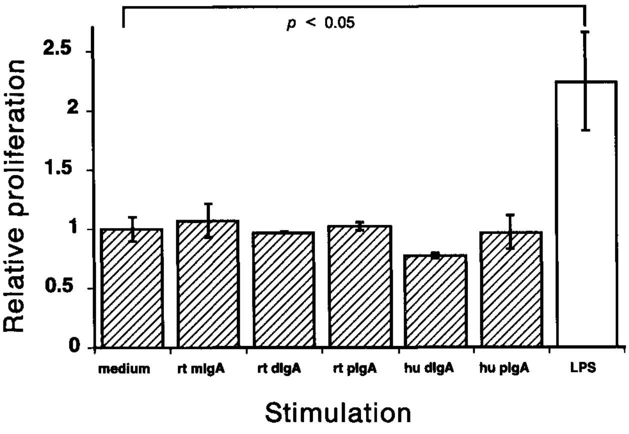

FIG. 5. Effect of various preparations of IgA on the proliferation of MC *in vitro*. MC were incubated with 10 μg/ml of various IgA preparations and LPS, as a positive control. The relative proliferation is defined as the mean number of cells per well incubated for 7 days with IgA or LPS, divided by the mean number of cells per well incubated for 7 days with medium alone. Results are expressed as the mean ± S.E.M. of triplicate wells.

### References

1. Mestecky J, McGhee JR. Immunoglobulin A (IgA): molecular and cellular interactions involved in IgA biosynthesis and immune response. *Adv Immunol* 1987; **40**: 153–245.
2. Heremans JF. Immunoglobulin A. In: Sela M, ed. *The Antigens, Volume 2*. New York: Academic Press, 1974; 365–522.
3. Conley ME, Cooper MD, Michael AF. Selective depositions of immunoglobulin A1 in immunoglobulin A nephropathy, anaphylactoid purpura nephritis and systemic lupus erythematosis. *J Clin Invest* 1980; **66**: 1432–1436.
4. Valentijn RM, Radl J, Haaijman JJ, *et al.* Circulating and mesangial secretory component-binding IgA1 in primary IgA nephropathy. *Kidney Int* 1984; **26**: 760–766.
5. Rodico JL. Idiopathic IgA nephropathy. *Kidney Int* 1984; **25**: 717–729.
6. van den Wall Bake AWL, Daha MR, van der Ark A, Hiemstra P, Radl J. Serum levels and *in vitro* production of IgA subclasses in patients with primary IgA nephropathy. *Clin Exp Immunol* 1988; **74**: 115–120.
7. van den Wall Bake AWL, Beyer WEP, Evers-Schouten JH, Daha MR, van Es LA. The humoral response to influenza vaccination in patients with primary immunoglobulin A nephropathy. An analysis of isotype distribution and size of the influenza-specific antibodies. *Am Soc Clin Invest* 1989; **84**: 1070–1075.
8. Rocatello D, Picciatto G, Coppo R. Clearance of polymeric IgA aggregates in humans. *Am J Kidney Dis* 1989; **14**: 354–360.

9. Gómez-Guerrero C, González E, Egido J. Evidence for a specific IgA receptor in rat and human mesangial cells. *J Immunol* 1993; **151**: 7172–7181.
10. Monteiro RC, Grossetête B, Nguyen AT, Jungers P, Lehuen A. Dysfunction of Fcα receptors by blood phagocytic cells in IgA nephropathy. *Contrib Nephrol* 1995; **111**: 116–122.
11. Kishimoto T. The biology of interleukin 6. *Blood* 1989; **74**: 1–10.
12. Horii Y, Muraguchi A, Iwano M, *et al.* Involvement of IL-6 in mesangial proliferative glomerulonephritis. *J Immunol* 1989; **143**: 3949–3955.
13. Sironi M, Breviario F, Proserpio P, *et al.* IL-1 stimulates IL-6 production in endothelial cells. *J Immunol* 1989; **142**: 549–553.
14. Kirnbauer R, Kock A, Schwarz T, *et al.* IFN-β-2, B cell differentiation factor 2, or hybridoma growth factor (IL-6) is expressed and released by human epidermal cells and epidemoid carcinoma cell lines. *J Immunol* 1989; **142**: 1922–1928.
15. Aarden LA, de Groot ER, Schaap OL, Lansdorp PM. Production of hybridoma growth factor by human monocytes. *Eur J Immunol* 1987; **17**: 1411–1416.
16. Bauer J, Ganter U, Geiger T, *et al.* Regulation of interleukin 6 expression in cultured human blood monocytes and monocyte-derived macrophages. *Blood* 1988; **72**: 1134–1140.
17. van Snick J, Cayphas S, Vink A, Uyttenhove C, Coulie PG, Rubira MR, Simpson RJ. Purification and $NH_2$-terminal amino acid sequence of a T-cell-derived lymphokine with growth factor activity for B-cell hybridomas. *Proc Natl Acad Sci USA* 1986; **83**: 9679–9683.
18. Gómez-Guerrero C, López-Armada MJ, González E, Egido J. Soluble IgA and IgG aggregates are catabolized by cultured rat mesangial cells and induce production of TNF-α and IL-6, and proliferation. *J Immunol* 1994; **153**: 5247–5255.
19. van den Dobbelsteen MEA, van der Woude FJ, Schroeijers WEM, van den Wall Bake AWL, van Es LA, Daha MR. Binding of dimeric- and polymeric IgA to rat renal mesangial cells enhances the release of interleukin 6. *Kidney Int* 1994; **46**: 512–519.
20. van den Dobbelsteen MEA, van der Woude FJ, Schroeijers WEM, van Es LA, Daha MR. Soluble aggregates of IgG and immune complexes enhance the production of IL-6 by renal mesangial cells. *Kidney Int* 1993; **43**: 544–553.
21. Reuf C, Budde K, Lacy J, Northemann W, Baumann M, Sterzel RB, Coleman DL. Interleukin 6 is an autocrine growth factor for mesangial cells. *Kidney Int* 1990; **38**: 249–257.
22. Striker GE, Striker LJ. Biology of disease. Glomerular cell culture. *Lab Invest* 1985; **53**: 122–131.
23. Müller GA, Kim AE, Vernier GK, van der Hem GK, van der Woude, FJ. Explantation of mesangial cell 'hillocks': a method for obtaining human mesangial cells in culture. *Int J Exp Pathol* 1992; **73**: 9–20.
24. Hiemstra PS, Gorter A, Stuurman ME, van Es LA, Daha MR. Activation of the alternative pathway of complement by human serum IgA. *Eur J Immunol* 1987; **17**: 321–326.
25. Rits M, Cormont F, Bazin H, Meykens R, Vaerman JP. Production of IgA secreting hybridomas with specificity for the 2,4-dinitrophenyl (DNP) hapten. *J Immunol* 1986; **89**: 81–87.
26. Helle M, Boeije L, Aarden LA. Functional discrimination between interleukin 6 and interleukin 1. *Eur J Immunol* 1988; **18**: 1535–1540.
27. van den Dobbelsteen MEA, van der Woude FJ, Schroeijers WEM, Klar Mohamad N, van Es LA, Daha MR. Clq, a subunit of the first component of complement, enhances the binding of aggregated IgG to rat renal mesangial cells. *J Immunol* 1993; **151**: 4315–4324.
28. Santiago A, Satriano J, De Candidio S, Holthoffer H, Schreiber R, Unkeless J, Schlondorff D. A specific Fcγ receptor on cultured rat mesangial cells. *J Immunol* 1989; **143**: 2575–2582.
29. Davies M. The mesangial cell: a tissue culture view. *Kidney Int* 1994; **45**: 320–327.
30. Floege J, Topley N, Hoppe J, Barrett TB, Resch K. Mitogenic effect of platelet-derived growth factor in human glomerular mesangial cells: modulation and/or suppression of inflammatory cytokines. *Clin Exp Immunol* 1991; **86**: 334–341.
31. Suematsu S, Matsuda M, Aozasa K, *et al.* IgG1 plasmacytosis in interleukin 6 transgenic mice. *Proc Natl Acad Sci* 1989; **86**: 7547–7551.
32. Gordon C, Richards N, Howie AJ, Richardson K, Michael J, Adu D, Emery P. Urinary IL-6: a marker for mesangial proliferative glomerulonephritis? *Clin Exp Immunol* 1991; **86**: 145–149.

**Received 3 January 1996; accepted 22 February 1996**
